# Oral mucous membrane pemphigoid as a window to systemic autoimmunity: two cases associated with rheumatoid arthritis and Sjögren's disease

**DOI:** 10.31744/einstein_journal/2026RC2514

**Published:** 2026-10-08

**Authors:** Isabella Castro de Oliveira, Laura Silva dos Santos, Jessica dos Santos Rodrigues, Renan Rodrigues Neves Ribeiro do Nascimento, Laura Caldas dos Santos, Juliana Mota Siqueira

**Affiliations:** 1 Hospital Israelita Albert Einstein Faculdade Israelita de Ciências da Saúde Albert Einstein São Paulo SP Brazil Faculdade Israelita de Ciências da Saúde Albert Einstein, Hospital Israelita Albert Einstein, São Paulo, SP, Brazil.; 2 Universidade Federal de São Paulo Escola Paulista de Medicina Department of Rheumatology São Paulo SP Brazil Department of Rheumatology, Escola Paulista de Medicina, Universidade Federal de São Paulo, São Paulo, SP, Brazil.; 3 Universidade Federal de São Paulo Escola Paulista de Medicina Department of Ophthalmology and Visual Sciences São Paulo SP Brazil Department of Ophthalmology and Visual Sciences, Escola Paulista de Medicina, Universidade Federal de São Paulo, São Paulo, SP, Brazil.

**Keywords:** Autoimmune diseases, Pemphigoid, benign mucous membrane, Arthritis, rheumatoid, Sjögren's disease, Diagnosis, oral

## Abstract

Mucous membrane pemphigoid is a rare chronic autoimmune bullous disease that predominantly affects mucosal surfaces and may lead to significant morbidity when diagnosis is delayed. Increasing evidence suggests that oral manifestations may serve as reliable early indicators of systemic immune dysregulation and can precede the recognition of other autoimmune disorders. We report two cases of oral mucous membrane pemphigoid associated with systemic autoimmune diseases, emphasizing that the oral cavity is a sentinel site for systemic autoimmunity and highlighting the importance of integrated diagnostic and therapeutic strategies. In the first case, a 68-year-old woman with longstanding rheumatoid arthritis presented with recurrent oral ulcerations that were histopathologically confirmed as mucous membrane pemphigoid. During follow-up, ocular involvement was identified as peripheral ulcerative keratitis related to rheumatoid arthritis, requiring adjustment of systemic immunosuppressive therapy. Switching to B-cell depletion therapy with rituximab resulted in the control of both the extra-articular rheumatoid arthritis manifestation and mucous membrane pemphigoid activity. In the second case, a 44-year-old woman presented with desquamative gingivitis and oral bullous lesions. After a biopsy confirmed mucous membrane pemphigoid, progressive sicca symptoms prompted rheumatological investigation, leading to the diagnosis of Sjögren's disease based on serology, imaging, and minor salivary gland biopsy. Treatment with azathioprine resulted in the clinical stabilization of both diseases. These cases illustrate how oral lesions may reveal or precede systemic autoimmune disease and underscore the diagnostic challenges of overlapping immune-mediated rheumatic diseases. Recognizing shared immunopathogenic pathways may enable the selection of targeted therapies that simultaneously control multiple autoimmune conditions, thereby improving disease control and reducing the treatment burden.

## INTRODUCTION

Mucous membrane pemphigoid (MMP) is a chronic autoimmune bullous disease with a predilection for mucosal surfaces, where it may cause pain, scarring, and significant functional impairment.^(1)^ It is characterized predominantly by mucosal lesions that commonly affect the oral, ocular, nasal, and anogenital mucosae,^(1,2)^ and delayed diagnosis may result in irreversible sequelae.

Mucous membrane pemphigoid mostly occurs in isolation, but its coexistence with systemic autoimmune diseases has been increasingly reported. However, associations with rheumatoid arthritis and Sjögren's disease remain rare and poorly characterized, being largely confined to isolated case reports and small case series.^(3–5)^ From a clinical and translational standpoint, these associations are highly relevant, as MMP-related mucosal lesions may precede systemic manifestations, mimic more common inflammatory or sicca-related disorders, or be misattributed to treatment-related adverse effects, ultimately leading to delayed diagnosis and suboptimal therapeutic interventions.

The coexistence of MMP and rheumatoid arthritis poses specific diagnostic challenges, particularly when extra-articular manifestations and overlapping clinical features are present. Similarly, Sjögren's disease may occur concomitantly with MMP, and its sicca-related oral and ocular involvement can obscure or simulate early signs of mucosal blistering disease, further complicating timely recognition.^(6,7)^ Although these conditions arise from distinct immunopathological mechanisms, growing evidence suggests potential immunological crosstalk that may contribute to their concomitant presentation, underscoring the need for an integrated, multidisciplinary diagnostic and therapeutic approach.^(8)^

In routine practice, immunosuppressive regimens are usually tailored to the predominant autoimmune disease; nevertheless, in overlap scenarios, a more integrated, targeted strategy capable of simultaneously inducing remission across coexisting autoimmune conditions is desirable. To illustrate these diagnostic and therapeutic challenges, we present two cases in which oral MMP manifestations guided subsequent diagnostic and therapeutic decision-making.

## CASE REPORT

### CASE 1

A 68-year-old female patient was referred for oral evaluation because of recurrent oral ulcerations persisting for 1 year, with no clinical remission despite previous photobiomodulation and topical medications prescribed by her general dentists. Her medical history was significant for rheumatoid arthritis, generalized anxiety disorder, chronic obstructive pulmonary disease, and atrial fibrillation. Current medications included tocilizumab and methotrexate (10mg weekly). Initial intraoral examination demonstrated multiple ulcerative lesions with a fibrinous base and a surrounding erythematous halo associated with diffuse epithelial fragility (Figure 1A-B). The patient reported that the lesions initially manifested as blisters before evolving into ulcerations, leading to a primary clinical suspicion of autoimmune bullous disease, particularly pemphigus vulgaris and MMP. Moreover, the long-term use of low-dose methotrexate without prior adverse events, appropriate folic acid supplementation, absence of neutropenia on complete blood count, and clinical progression to bullous lesions made methotrexate-induced mucositis unlikely at that stage. An incisional biopsy of the buccal mucosa was performed under local anesthesia, and histopathological examination demonstrated subepithelial clefting in the basement membrane zone, supporting the diagnosis of MMP. Initial management included topical dexamethasone to control oral lesions during disease flares.

**Figure 1:**
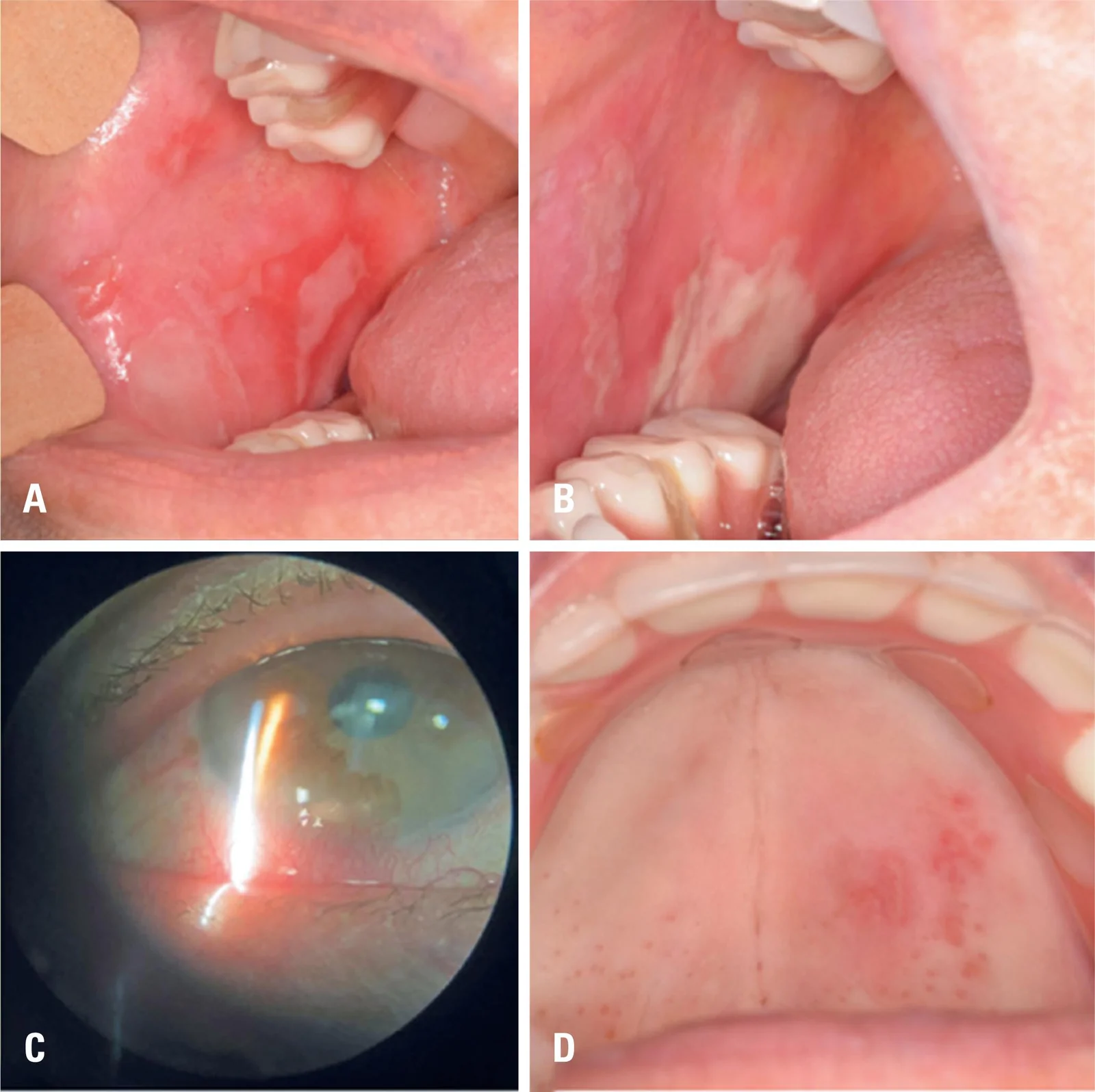
A-B) Examination of the oral cavity revealed diffuse erythema of the marginal gingiva with a smooth, friable surface, areas of epithelial desquamation, and irregular erosions covered by whitish–yellowish pseudomembranes on the buccal mucosa. C) Ophthalmological evaluation showing a peripheral inflammatory infiltrate without thinning, consistent with early peripheral ulcerative keratitis. D) Multiples punctate palatal ulcerations, suggestive of secondary herpes simplex virus infection

After 1 year of follow-up, the patient's clinical symptoms had significantly worsened, with an increased number of oral ulcerations, xerostomia, lesions suggestive of secondary herpes simplex virus (HSV) infection, and newly developed ocular involvement (Figure 1C). Intraoral examination demonstrated ulcerative lesions covered by a darker fibrinous membrane, in addition to multiple punctate palatal ulcerations, suggestive of secondary HSV infection (Figure 1D). Antimicrobial photodynamic therapy was used as an adjunctive treatment for the suspected secondary HSV infection.^(9,10)^

The patient was referred to the ophthalmology and rheumatology departments for comprehensive multidisciplinary evaluation. The ocular findings included an oval infiltrate in the corneal stroma, irregular epithelial loss in the area adjacent to the limbus, and vasodilation of scleral vessels in the quadrant corresponding to the corneal lesion, as observed in precursor lesions of peripheral ulcerative keratitis (PUK). This biomicroscopic finding suggested the reactivation of rheumatoid arthritis. Given the refractoriness of the disease to tocilizumab and progression to ocular involvement consistent with early PUK secondary to rheumatoid arthritis, the therapeutic regimen was revised. Tocilizumab was discontinued and replaced with rituximab to address the extra-articular manifestation of rheumatoid arthritis. Peripheral CD19^+^ B-cell levels were monitored before and after B-cell depletion, methotrexate was maintained, and short-term systemic corticosteroid therapy was introduced. At 6-month follow-up, the patient demonstrated disease control.

### CASE 2

A 44-year-old female patient was initially evaluated by an oral medicine specialist for painful gingival inflammation and oral blistering that developed following dental restorative treatment. Intraoral examination showed diffuse marginal gingival erythema with a smooth, friable surface and areas of epithelial desquamation on the buccal mucosa (Figure 2A-B). Nikolsky's sign was positive. Incisional biopsy revealed a subepithelial inflammatory process with subepithelial clefting in the basement membrane zone, consistent with MMP. Prednisone (10mg/day) produced partial, transient improvement but was discontinued due to gastrointestinal adverse effects.

**Figure 2:**
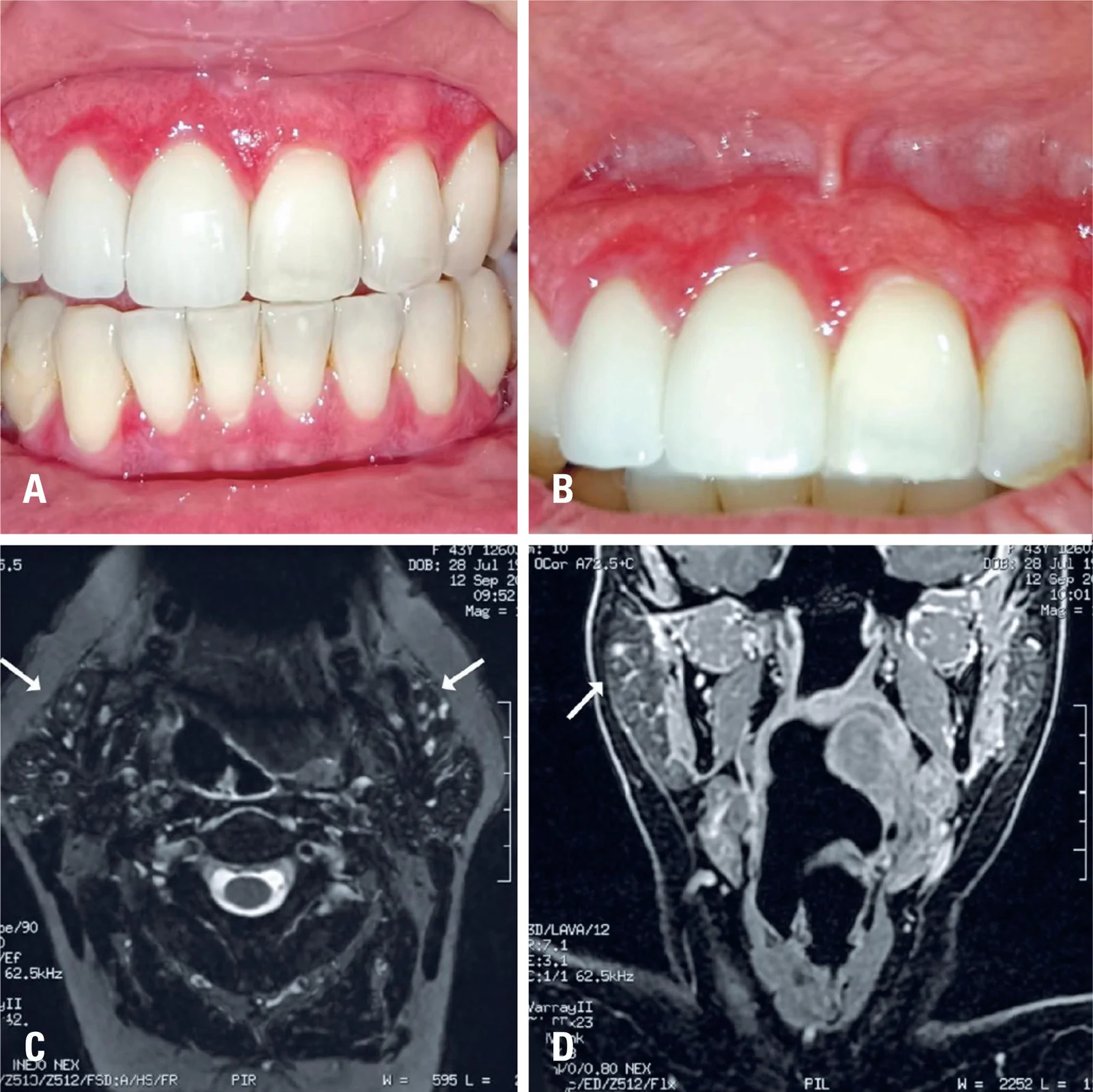
A and B) Oral cavity examination showing diffuse erythema of the marginal gingiva, with a smooth, friable surface and areas of epithelial desquamation on the buccal mucosa. C and D) Magnetic resonance imaging demonstrating irregular parotid glands; on T1-weighted images, there is bilateral fatty replacement of the parotid glands with interspersed hyperintense areas (calcifications), yielding a characteristic "salt-and-pepper" appearance (arrow) indicative of Sjögren's disease

Over the subsequent months, the patient developed persistent xerostomia and ocular discomfort, along with a recurrent oral burning sensation and bilateral parotid enlargement. Given these sicca symptoms and complaints of fatigue, she was referred to a rheumatologist for evaluation of suspected Sjögren's disease. A diagnostic workup revealed high-titer anti-Ro/SSA and a positive antinuclear antibody test with a fine-speckled nuclear pattern. Magnetic resonance imaging findings showed bilateral fatty replacement of the parotid glands with interspersed hyperintense areas (calcifications), yielding a characteristic "salt-and-pepper" appearance (Figure 2C-D) indicative of Sjögren's disease. A minor salivary gland from the lower lip biopsy showed focal lymphocytic sialadenitis with a focus score of 1.23/4mm², acinar atrophy, and focal lymphoepithelial lesions, fulfilling the diagnostic criteria for Sjögren's disease.

Hydroxychloroquine (400mg/day) was initiated to manage the systemic manifestations; however, treatment was stopped because of severe diarrhea and headache, followed by worsening MMP activity. Given the coexistence of MMP and Sjögren's disease, azathioprine was introduced as a steroid-sparing immunosuppressive therapy and titrated to 200mg/day. Using a combined systemic approach and supportive topical measures, including temporary use of silicone trays for medicament delivery to control gingival symptoms,^(11)^ both diseases stabilized, with fewer MMP flares and better control of sicca symptoms. At the 20-month follow-up, the patient remained clinically stable with adequate disease control.

The article was approved by the Research Ethics Committee of *Faculdade de Odontologia, Universidade de São Paulo* (CAAE: 00759926.3.0000.0075; #8.698.308). Patient consent to participate was obtained.

## DISCUSSION

Mucous membrane pemphigoid is characterized by chronic, scarring inflammation of mucosal surfaces, but its clinical expression may be modified or obscured in patients who already have other systemic autoimmune diseases.^(12)^ Overlap with conditions such as rheumatoid arthritis and Sjögren's disease has been described only in isolated reports and small series; therefore, the frequency, spectrum, and prognostic implications of these associations remain poorly defined.^(3–5)^

These reports describe patients with longstanding rheumatoid arthritis in whom articular disease consistently preceded the onset of MMP by several years or even decades. Oral erosions, desquamative gingivitis, and ocular involvement, particularly symblepharon and conjunctival scarring, were the predominant manifestations, and most patients required escalation of systemic immunosuppressive therapy beyond corticosteroids, reflecting the complexity of this overlap syndrome.^(3–5)^

Our first case shares several of these features, including advanced age, longstanding rheumatoid arthritis, delayed onset of oral MMP, and the need for adjustment of systemic therapy. However, unlike previously reported cases, the ocular involvement was attributed to rheumatoid arthritis-associated PUK rather than MMP, highlighting the importance of distinguishing overlapping autoimmune manifestations, as this distinction directly influenced therapeutic decision-making.

Reports of MMP associated with Sjögren's disease are even more limited, precluding any meaningful estimate of incidence given the rarity of published cases. The only report we identified described a 36-year-old woman with a history of thymoma who initially presented with xerostomia and oral candidiasis, followed by ocular surface disease attributed to Sjögren's disease. The diagnosis of Sjögren's disease was established based on positivity for anti-SS-A/SS-B antibodies and a labial salivary gland biopsy revealing focal lymphocytic sialadenitis. Oral MMP, confirmed by biopsy and direct immunofluorescence, developed only several months later, after immunosuppressive therapy for Sjögren's disease had already been initiated, which was the reverse of the sequence observed in our second patient.^(13)^

In contrast to the rheumatoid arthritis-associated cases, our second patient initially presented with biopsy-confirmed oral MMP, while persistent xerostomia, ocular discomfort, recurrent parotid enlargement, and supportive laboratory and histopathological findings subsequently led to a diagnosis of Sjögren's disease. This reversed temporal sequence (MMP preceding the systemic diagnosis) suggests that oral MMP may precede the recognition of systemic autoimmune disease and reinforces the role of the oral cavity as a potential early site of clinically relevant immune dysregulation.

Taken together, these cases expand the limited literature on MMP overlap syndromes and highlight the importance of multidisciplinary assessment for timely diagnosis and appropriate therapeutic management.

In rheumatoid arthritis, oral involvement is frequently under-recognized and may manifest as temporomandibular joint dysfunction and synovitis, as well as xerostomia, burning mouth, and oral ulcerations, particularly in patients undergoing methotrexate therapy.^(14,15)^ Similarly, Sjögren's disease usually presents with xerostomia and keratoconjunctivitis sicca, but may also lead to burning or sore mouth, dysgeusia, difficulty retaining dentures, cervical and root caries, candidiasis, and recurrent parotid enlargement.^(16,17)^ Many of these features overlap with those of MMP, particularly when chronic erosions and erythematous mucositis are more prominent than intact blisters, increasing the risk of diagnostic delay or misclassification as another ulcerative disease, such as erosive oral lichen planus and pemphigus vulgaris.^(18)^ In this context, meticulous oral examination, a high index of suspicion, and timely biopsy remain crucial to distinguish disease-specific patterns from non-specific inflammatory changes.^(19)^Table 1 summarizes the more typical and less common oral findings in rheumatoid arthritis, Sjögren's disease, and MMP, highlighting points of convergence and divergence that may assist clinicians in daily practice.

**Table 1:** Comparative oral and maxillofacial manifestations in mucous membrane pemphigoid, rheumatoid arthritis, and Sjögren's disease

Disease	Classic clinical features	Less common / rare clinical features	Key laboratory / histopathologic markers	Treatment
Oral mucous membrane pemphigoid	– Blistering lesion; – Desquamative gingivitis; – Erythematous and easily bleeding gingiva; – Tense bullae; – Painful erosions on the buccal mucosa, palate, and tongue; – Burning oral sensation; – Positive Nikolsky sign	– Nasopharyngeal, oropharyngeal, laryngeal, and esophageal involvement; – Dysphagia; – Dysphonia; – Obstructive airway compromise	– Subepithelial clefting with mixed inflammatory infiltrates (lymphocytes, eosinophils, neutrophils) and subepithelial fibrosis; – DIF: linear deposition of IgG, IgA, and/or C3 along the basement membrane zone; – IIF/ELISA: autoantibodies to BP180, BP230, laminin-332 or α6β4 integrin	Topical: – High-potency CS; – Calcineurin inhibitors; Systemic: – Dapsone or tetracyclines; – CS – Methotrexate; – Mycophenolate mofetil; – Cyclophosphamide – Immunoglobulin – Rituximab
Rheumatoid arthritis	– TMJ involvement with jaw pain, stiffness, reduced mouth opening, crepitus, and malocclusion; – Xerostomia and burning mouth, with or without SD	– Cricoarytenoid arthritis causing hoarseness, throat pain, odynophagia, dysphonia, dyspnea or stridor, and rarely acute airway obstruction; – Rheumatoid nodules in the larynx or oral cavity; – Vasculitic oral ulcers; – Secondary amyloidosis of the salivary glands	Serology: RF and anti-CCP antibodies, anti-CarP. Elevated ESR and/or CRP; Imaging: The TMJ may show erosions and deformity on radiographs, MRI, or ultrasound in longstanding disease	Topical: – TMJ disease: occlusal splints, physiotherapy, and intra-articular CS injections; – Cricoarytenoid disease: CS injection and tracheostomy in life-threatening obstruction Systemic: – Methotrexate, LFM – CS – Biologic DMARDs or targeted synthetic DMARDs
Sjögren's disease (primary or secondary)	– Xerostomia; – Dysphagia; – Burning or sore mouth; – Dysgeusia and difficulty retaining dentures. – Cervical/root caries; – Oral candidiasis. – Recurrent or persistent swelling of parotid and/or submandibular glands	– Salivary gland lymphoma (often MALT-type); – Suppurative sialadenitis with sialectasia; – Cranial neuropathies (e.g., trigeminal); – Small-fiber neuropathy; – Vasculitic skin ulcers	Serology: ANA, anti-Ro-52/SSA, anti-La/SSB, and RF positive. Elevated serum IgG. Cryoglobulinemia and low C4 levels. Objective tests include Schirmer's test, OSS, unstimulated whole salivary flow, and salivary gland USG with hypoechoic lesions and calcifications in the parenchyma Minor salivary gland (labial) biopsy shows focal lymphocytic sialadenitis with a focus score ≥1 focus/4 mm².	Topical: – Sugar-free chewing gum/lozenges -saliva substitutes. – Cholinergic agonists (pilocarpine, cevimeline, bethanechol chloride); Systemic disease: – Hydroxychloroquine – GS; – Methotrexate; – Azathioprine; – Rituximab

ANA: antinuclear antibody; anti-CCP: anti–cyclic citrullinated peptide antibody; anti-CarP: anti–carbamylated protein antibody; BP180: bullous pemphigoid antigen 180; BP230: bullous pemphigoid antigen 230; C3: complement component 3; C4: complement component 4; CRP: C-reactive protein; CS: corticosteroids; DIF: direct immunofluorescence; DMARDs: disease-modifying antirheumatic drugs; ELISA: enzyme-linked immunosorbent assay; ESR: erythrocyte sedimentation rate; GS: systemic glucocorticoids; IgA: immunoglobulin A; IgG: immunoglobulin G; IIF: indirect immunofluorescence; LEF: leflunomide; MALT: mucosa-associated lymphoid tissue; MRI: magnetic resonance imaging; OSS: ocular staining score; RF: rheumatoid factor; SD: Sjögren's disease; SSA: Ro/SSA antibodies; SSB: La/SSB antibodies; TMJ: temporomandibular joint; USG: ultrasonography.

Our cases illustrate two complementary diagnostic scenarios. In the first case, oral MMP developed in a patient with longstanding rheumatoid arthritis who was receiving biologic therapy, and subsequent ocular involvement raised the possibility of pemphigoid-related eye disease. However, a detailed ophthalmological assessment ultimately identified PUK as an extra-articular manifestation of rheumatoid arthritis rather than MMP. This underscores how, in overlap settings, attributing all mucosal and ocular findings to a single autoimmune process may be misleading and may delay the appropriate adjustment of rheumatoid arthritis-directed immunosuppression. In the second case, desquamative gingivitis and blistering oral lesions were the initial presentations, leading to the diagnosis of MMP and prompting a systematic workup for sicca symptoms, culminating in the recognition of underlying Sjögren's disease through serology, salivary gland biopsy, and characteristic imaging findings.^(13)^ In this scenario, oral findings represented an early clinical manifestation of occult systemic autoimmunity, enabling prompt rheumatological investigation and multidisciplinary management.

A pathophysiological understanding of each overlapping disease is essential, particularly in an era in which several targeted agents modulate distinct yet intersecting immune pathways. In rheumatoid arthritis and Sjögren's disease, B-cell hyperactivity and autoantibody production are central features, whereas in MMP, pathogenic autoantibodies directed against components of the basement membrane zone drive subepithelial blister formation. Although the primary mechanisms differ, therapies targeting B cells, T-cell co-stimulation, or key cytokine axes (such as interleukin-6) may exert beneficial effects across multiple autoimmune diseases.^(20)^ In our rheumatoid arthritis-MMP case, switching from interleukin-6 receptor blockade (tocilizumab) to B-cell depletion with rituximab in response to PUK reflects a strategy to control both articular and ocular vasculitic activity while maintaining adequate MMP control. In the MMP-Sjögren's disease case, azathioprine provided a steroid-sparing option that stabilized both MMP activity and Sjögren's disease symptoms. Azathioprine may be effective in both conditions (MMP-Sjögren's disease) by inhibiting T- and B-lymphocyte proliferation through the suppression of purine synthesis, thereby reducing autoantibody production and tissue inflammation. Figure 3 schematically summarizes the core pathogenic pathways implicated in rheumatoid arthritis, Sjögren's disease, and MMP, and illustrates how they can be targeted within an integrated therapeutic framework.

**Figure 3:**
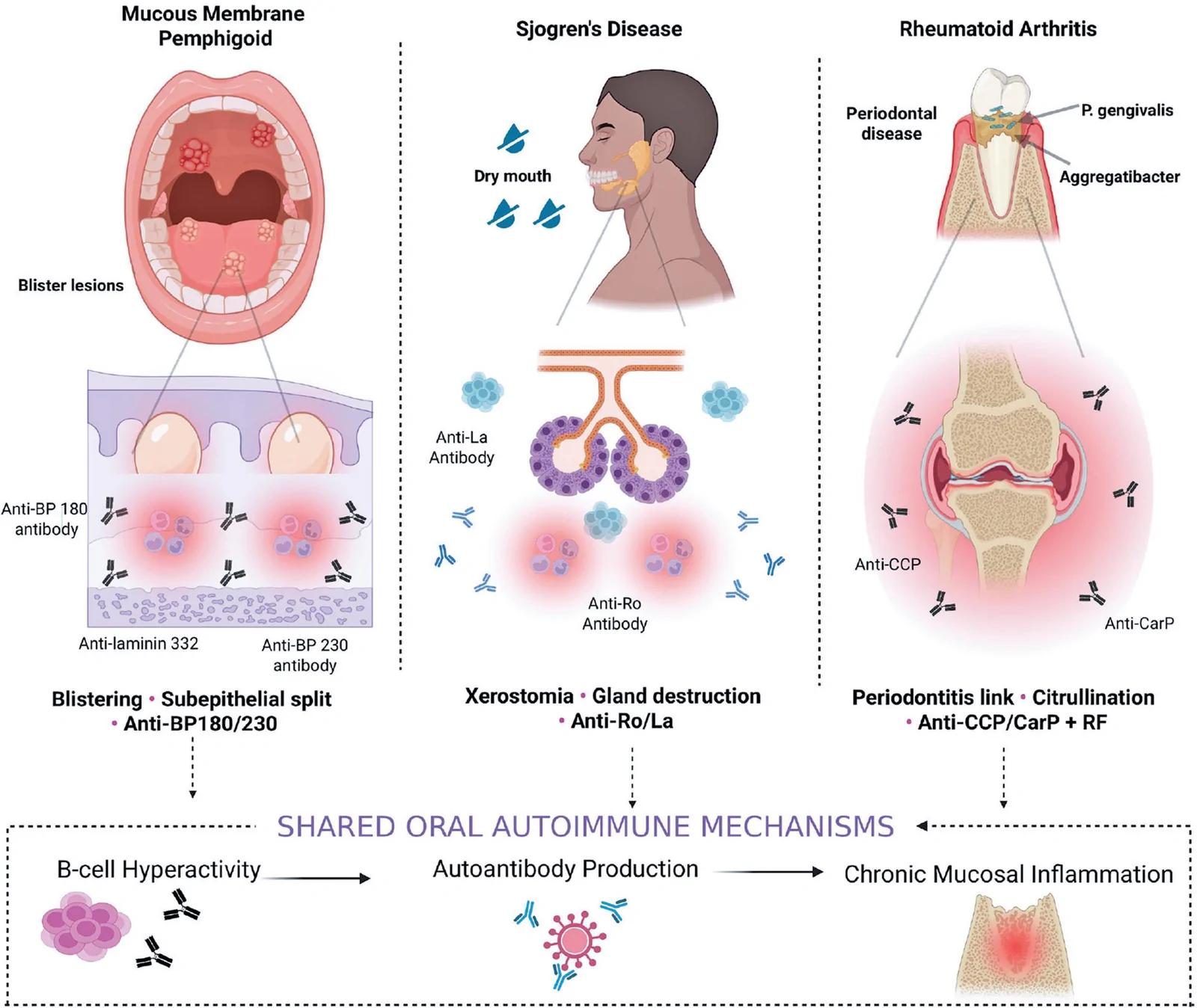
Pathophysiological mechanisms of oral involvement in mucous membrane pemphigoid, Sjögren's disease, and rheumatoid arthritis Schematic representation of the key immunopathogenic pathways linking these three autoimmune diseases to oral and maxillofacial manifestations. Left – Mucous membrane pemphigoid: autoantibodies against BP180/BP230, laminin-332, and the α6β4 integrin target components of the epithelial basement membrane zone, leading to complement activation, subepithelial separation, and formation of large, fragile mucosal blisters. Center – Sjögren's disease: salivary glands show dense periductal and intralobular lymphocytic infiltration, driven in part by anti-Ro/SSA and anti-La/SSB autoantibodies, with progressive acinar destruction, hyposalivation, and xerostomia. Right – Rheumatoid arthritis: periodontal dysbiosis involving *Porphyromonas gingivalis* and *Aggregatibacter actinomycetemcomitans* promotes protein citrullination and carbamylation, breaking tolerance and amplifying the production of rheumatoid factor, anti-CCP, and anti-CarP autoantibodies; these systemic autoantibody responses contribute to synovial inflammation and bone erosion

Source: created with the tool BioRender. Available from: https://www.biorender.com/

BP: bullous pemphigoid antigen; BP180/BP230: bullous pemphigoid antigens 180/230; CCP: cyclic citrullinated peptide; CarP: carbamylated protein; RF: rheumatoid factor; SSA: Ro antigen; SSB: La antigen.

This report has several limitations. It describes only two patients, reflecting both the rarity of the association and a potential referral bias towards more complex oral mucosal disease. The follow-up period was relatively short, which may have precluded the detection of late complications such as relapse, progressive ocular damage, or lymphoma in the setting of Sjögren's disease. Furthermore, although direct immunofluorescence is the gold standard for confirming autoimmune bullous disease, it was not performed because of limitations in accessibility and cost. Therefore, the absence of immunopathological confirmation represents a diagnostic limitation and reduces the certainty of the diagnosis. Nevertheless, the clinical presentations were supported by consistent clinical and histopathological findings. Despite these limitations, these cases highlight an uncommon clinical overlap and reinforce the role of the oral cavity as a potential sentinel site for systemic autoimmunity.

## CONCLUSION

These findings illustrate that oral mucous membrane pemphigoid can act as a sentinel manifestation of underlying or evolving systemic autoimmunity, including rheumatoid arthritis and Sjögren's disease. In patients with chronic erosive or blistering oral lesions, meticulous oral examination and timely biopsy are essential to unmask concomitant autoimmune diseases and distinguish disease-specific from overlapping features. Early recognition of these rare overlap scenarios allows immunosuppressive therapy to be tailored to shared pathogenic pathways while preserving oral and ocular function. These cases further underscore the importance of coordinated care among oral medicine, rheumatology, and ophthalmology. Larger series and multicenter studies are needed to refine prognostic assessment and to develop evidence-based therapeutic algorithms for patients with coexisting mucous membrane pemphigoid and systemic autoimmune disease.

## Data Availability

The underlying content is contained within the manuscript.
